# Synthesis and Characterization of a Marine Collagen–Chitosan/HA–SiO_2_-Based Bioink

**DOI:** 10.3390/gels12030197

**Published:** 2026-02-26

**Authors:** Andrea Cazares-Tafoya, Marcos Valenzuela-Reyes, Solange Rivera-Manrique, Carlos Martínez-Pérez, Odin Ramírez-Fernández, Esmeralda Zuñiga-Aguilar

**Affiliations:** 1 Department of Electrical Engineering and Computer Science, Autonomous University of Ciudad Juárez, Cd. Juárez 32320, Mexico; al192602@alumnos.uacj.mx (A.C.-T.); marcos.valenzuela@uacj.mx (M.V.-R.); 2 Department of Chemical, Electronic, and Biomedical Engineering, University of Guanajuato, León 37670, Mexico; si.rivera@ugto.mx; 3 Department of Physics and Mathematics, Institute of Engineering and Technology, Autonomous University of Ciudad Juárez, Cd. Juárez 32310, Mexico; camartin@uacj.mx; 4 Research Department, Universidad Tecnológica de México-UNITEC México-Campus en Línea, Col. Anáhuac, Ciudad de Mexico 11320, Mexico; odinramirezfernandez@upci.edu.mx

**Keywords:** bioprinting, bioink, marine collagen, chitosan, silica-doped hydroxyapatite

## Abstract

In this work, we report the synthesis and evaluation of a bioink based on marine collagen, chitosan, and silica-doped hydroxyapatite (HA–SiO_2_) for extrusion-based 3D bioprinting. FTIR spectroscopy confirmed amide (I–III) and phosphate/siloxane signals, TGA showed initial dehydration and degradation stages compatible with the process’s thermal handling, and SEM revealed an interconnected porous microstructure. Rheologically, the ink exhibited elastic dominance (G′ > G″) within the linear range and pseudoplastic, shear-thinning behavior—consistent with pneumatic extrusion. Process evaluation on a BIO X printer (14 G nozzle, low print speeds, moderate pressure, cartridge at 37 °C to 45 °C, and a cooled build platform) enabled deposition of strands with local shape retention. However, filament continuity was limited and line width varied, indicating only preliminary printability and a narrow operating window. Overall, physicochemical, microstructural, and rheological evidence supports the formulation’s viability as a starting point for scaffold fabrication.

## 1. Introduction

Extrusion bioprinting has become established as an additive manufacturing technology for generating three-dimensional scaffolds with controlled geometries and functional gradients, with applications in tissue engineering, in vitro modeling, and regenerative medicine [1]. The performance of the printed scaffold depends on the bioink’s ability to sustain a stable transition between rest and flow states and back again during the printing process, allowing the material to flow through the nozzle with moderate stress, and after deposition to quickly regain its structural integrity [2]. This transition is controlled by the chemical composition, thermal stability, microstructure, and critically the rheological response of the bioink under operating printing conditions [1].

Collagen is a biopolymer widely used for its biocompatibility and the presence of biochemical compounds that promote cell adhesion and signaling. However, its manipulation in extrusion printing (EP) presents challenges, including sensitivity to pH and temperature, environment-dependent gelation kinetics, and limited mechanical strength immediately after extrusion [3]. Combining it with chitosan, a cationic polysaccharide with electrostatic interactions and viscoelastic modulation capabilities, can increase viscosity at rest, promote structural recovery after shearing, and improve shape retention [4,5]. Similarly, hydroxyapatite (HA) provides osteoconductive functionality through doping with silica (HA–SiO_2_), modifying the surface energy and reactivity of the inorganic phase, facilitating interactions with the organic matrix, and contributing to the formation of stable porous networks, relevant attributes for bone applications in tissue engineering [6,7,8].

The concept of printability in EP consists of rheological and thermal properties. It considers the predominance of the elastic modulus over the viscous modulus (G′ > G″) in the linear range to maintain the shape after deposition, pseudoplastic behavior (shear thinning) [9] that reduces apparent viscosity during passage through the nozzle and limits extrusion pressure, the presence of sufficient yield strength or initial resistance to prevent collapse of the printed filament, and a thermal window that allows modulation of viscosity without compromising the chemical integrity of the material [9]. These properties must also be integrated with controllable process variables, such as nozzle diameter, travel speed, pneumatic pressure, and cartridge and print bed temperatures, to define a reproducible operating region that minimizes filament diameter variability and maximizes filament continuity [10,11].

In this work, the use of marine-derived collagen is proposed as an alternative to terrestrial sources, offering advantages in terms of supply and reproducibility, with the added benefit of mitigating regulatory, sanitary, or cultural objections associated with porcine materials. The protein source for biotin synthesis influences the transition temperature, thermal stability, and susceptibility to degradation. These factors directly affect the printing process, e.g., the thermal control strategy of the process, the use of a heated cartridge and a cold platform, as well as the dimensional stability of the scaffold. Chitosan contributes a positive charge and interaction points that modulate the viscosity at rest and structural recovery. The inclusion of a moderate HA–SiO_2_ fraction (approximately 1% *w*/*w*) enables exploration of synergies between bioactivity and microstructure control without affecting the material’s extrusion. Characterization by FTIR, TGA, and scanning electron microscopy (SEM) confirms the chemical identity, thermal compatibility with the process, and an interconnected porous microstructure. The rheological response shows elastic predominance in the linear range and pseudoplastic behavior in steady flow, attributes consistent with EP. Our proposal offers a viable formulation and methodology that connects material properties to printing parameters. At the same time, it clearly defines the need to optimize the experimental array to improve filament continuity and the dimensional stability of the resulting scaffolds.

## 2. Results and Discussion

### 2.1. Synthesis of Materials

**Figure 1:**
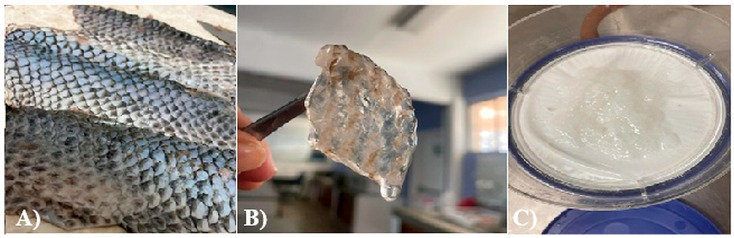
Collagen extraction: (**A**) Nile tilapia (*Oreochromis niloticus*) skin, (**B**) post-treatment with acetic acid, (**C**) marine collagen after dialysis.

The Nile tilapia (*Oreochromis niloticus*) skins used in the study initially appeared as shown in Figure 1A. The pre-freezing and thawing process shortened the tissue conditioning time, as the formation of ice crystals during freezing alters the internal microstructure and facilitates reagent penetration. Subsequently, after treatment with sodium hydroxide (NaOH), the skins exhibited a yellowish appearance, particularly when processed in an aqueous medium. Furthermore, the residual liquid acquired a similar color and an oily consistency, indicative of the removal of non-collagen components. Consistently with previous reports, this step facilitated the elimination of non-collagen proteins. After treatment with acetic acid, the skins acquired the appearance shown in Figure 1B, with a gelatinous consistency and greater transparency, although some residual pigmentation was still observed. Finally, Figure 1C shows the final product of the process: a paste with a lumpy, gelatinous consistency, which was characterized by FTIR spectroscopy to confirm its composition [12,13,14].

### 2.2. Chemical Composition

**Figure 2:**
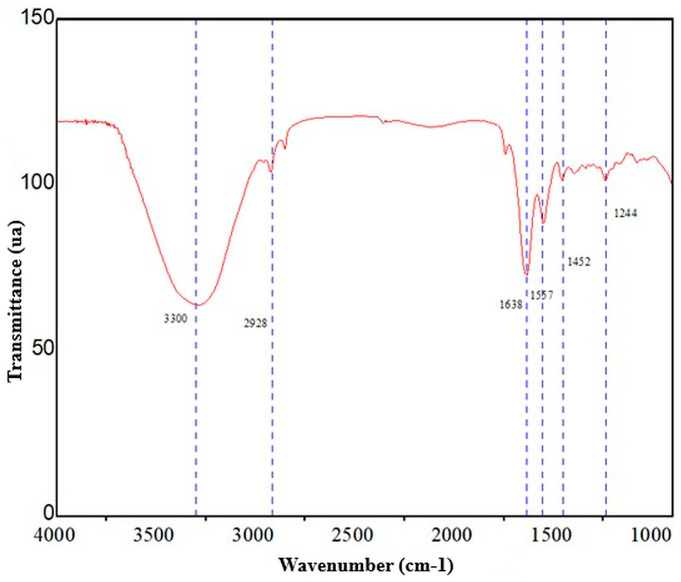
FTIR spectrum of marine collagen.

To confirm the chemical composition of the extracted material, FTIR spectroscopy was performed, and characteristic signals of amides A, B, I, II, and III, consistent with collagen, were identified. The spectrum in Figure 2 shows that the vibration associated with N–H stretching is located in the range of 3400 cm^−1^ to 3440 cm^−1^, but that the formation of hydrogen bonds with the N–H group decreased the frequency, with amide A located around 3300 cm^−1^, indicating the combination of N–H stretching with hydrogen bonding. Likewise, a weak signal attributed to amide B was observed at 2928 cm^−1^, corresponding to the asymmetric stretching of CH_2_. Regarding the most structurally significant bands, amide I was observed at 1638 cm^−1^ (C=O stretching), amide II at 1557 cm^−1^ (N–H bending), and amide III at 1244 cm^−1^ (related to vibrations of the peptide backbone), all of which are linked to the triple-helix conformation characteristic of collagen. To support structural conservation, the reported criterion used was that an absorption ratio close to 1 between the amide III band and the 1450–1454 cm^−1^ region suggests that the triple helix remains intact. In this case, a band at 1452 cm^−1^ with an approximate intensity of 100 was observed, thus inferring that the collagen structure remained intact after acid hydrolysis. The simultaneous presence of amides A and B and bands I–III, along with the criterion of the amide III ratio at 1450 cm^−1^ to 1454 cm^−1^, confirmed that the product obtained did not correspond to a nonspecific protein fraction, but rather to a matrix with a vibrational signature compatible with collagen and with preservation of features associated with the triple helix. It was confirmed that collagen was successfully extracted while maintaining structural characteristics relevant to its subsequent use as a polymeric component in hydrogel formulations [13,14,15,16].

**Figure 3:**
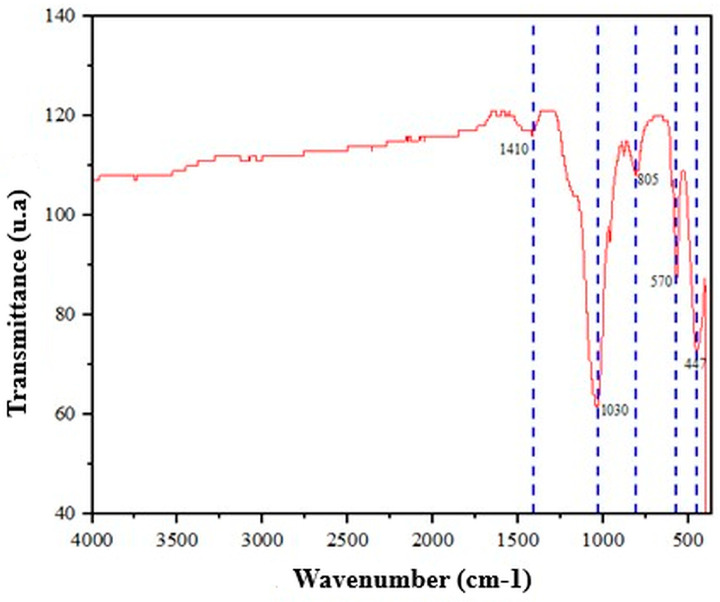
FTIR spectrum of silica-doped hydroxyapatite.

The silica-doped hydroxyapatite received from the University of Guanajuato was analyzed by FTIR spectroscopy to verify its composition. The spectrum in Figure 3 confirmed bands associated with the phosphate ion (PO_4_^3−^), with a bending mode around 570 cm^−1^ and an asymmetric stretching mode near 1030 cm^−1^, in addition to a band attributed to carbonate around 1410 cm^−1^, identified as hydroxyapatite. Furthermore, bands linked to the presence of silica were identified around 800 cm^−1^ (Si–O–Si bond bending) and near 450 cm^−1^ (Si–O oscillation). The same analysis indicated the absence of OH groups typically observed around 3000 cm^−1^, attributed to the heat treatment at 600 °C used during synthesis to reduce impurities from the manufacturing process. Based on the coexistence of functional signals from both components (hydroxyapatite and silica), an interaction between SiO_2_ and hydroxyapatite was confirmed, and substitutional doping (silicon–calcium) was inferred, supported by hydroxyapatite’s capacity for ionic substitution. The confirmation of PO_4_^3−^ and carbonate allowed us to conclude that the powder retained the chemical identity of a hydroxyapatite-like phase. The Si–O–Si and Si–O bands supported the incorporation of silica into the doped material. The absence of OH, interpreted in light of the 600 °C heat treatment, reinforced the idea of material purification/stabilization and helped explain spectral differences relative to untreated HA [17,18,19,20].

The selection of the C3–HS1 combination within the experimental matrix was based on two stages: first, C3 (85% chitosan–15% collagen) was selected as the base formulation. Subsequently, the level of functionalization with silica-doped hydroxyapatite was defined. This selection was justified by comparing the evaluated formulations: C3 exhibited more suitable rheological behavior for continuing the study. Previous formulations (C1, C2, C4, and C5) were discarded due to inconsistent viscosity results for the extrusion objective. In the second stage, among the materials C3–HS1 to C3–HS5, C3–HS1 was chosen because it showed the greatest extrusion behavior (a tendency toward stable flow and therefore greater extrusion viability within the equipment’s operational limitations) [21,22,23,24].

**Figure 4:**
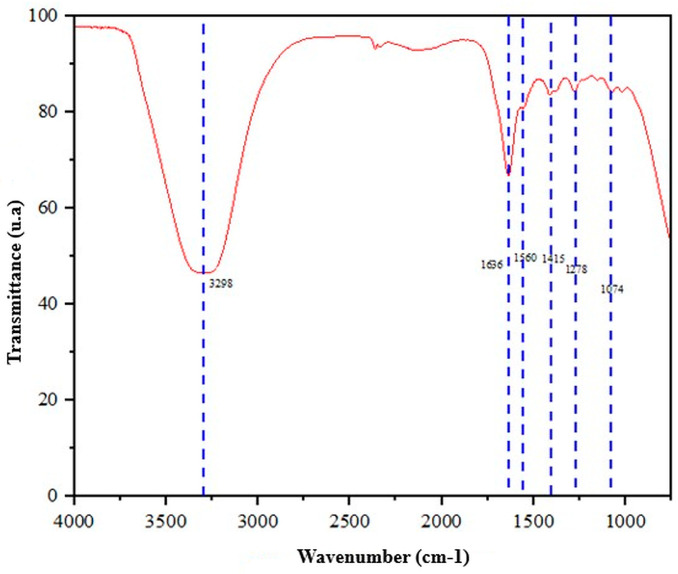
FTIR spectrum of the marine collagen–chitosan bioink doped with hydroxyapatite and silica.

To validate the integration of components in the selected formulation, an FTIR spectrograph study was performed on the C3–HS1 sample shown in Figure 4. A high-intensity band was observed around 3300 cm^−1^, which was interpreted as the combined contribution of OH groups (3000–3400 cm^−1^) and the N–H stretch. In the amide region, signals corresponding to amide I (C=O stretch), amide II at 1636 cm^−1^, and amide III at 1278 cm^−1^ were observed, associated with the protein and polysaccharide fractions of the system. Consistently with the incorporation of the ceramic phase, a band at 1074 cm^−1^ attributed to PO_4_^3−^ stretching and another at 1415 cm^−1^ corresponding to carbonate stretching were identified, confirming the presence of hydroxyapatite in the hydrogel. Furthermore, a band at 447 cm^−1^, associated with the silicon-oxygen oscillation, was observed, supporting the presence of the silica component in the functionalized formulation. It was also confirmed that the interaction between collagen and chitosan, represented by the shoulder band at 3362 cm^−1^, corresponded to O–H stretching from hydroxyl groups in chitosan and collagen enhanced by hydrogen bonding between the polymers [25], which would have favored the formation of a hybrid polymer network with reversible, hydrogel-like behavior. The coexistence within the same spectrum of signals from amides (a component of collagen), bands associated with hydroxyl and amine groups, and PO_4_^3−^/carbonate signatures (HA phase) alongside Si–O (silica) supported the idea that the formulation was not a simple physical mixture without chemical evidence, but rather a system where spectral fingerprints of each constituent were preserved within the final hydrogel. Furthermore, the explanation based on hydrogen bonding between collagen and chitosan provided a plausible mechanism for forming a continuous polymer matrix capable of incorporating a doped ceramic phase, reinforcing the hybrid network interpretation required for a structurally cohesive bioink [17,18,19,20].

### 2.3. Rheological Study

**Figure 5:**
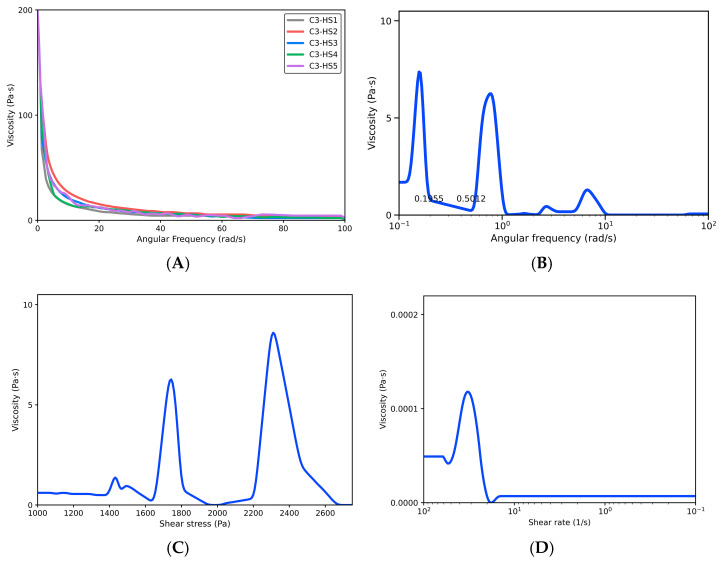
(**A**) Dynamic viscosity test as a function of angular frequency of collagen–chitosan bioink samples doped with hydroxyapatite and silica. (**B**) Frequency sweep test of C3–HS1. (**C**) Shear stress test versus dynamic viscosity of C3–HS1. (**D**) Shear rate test versus dynamic viscosity of C3–HS1.

The rheological results of the SiO_2_-doped collagen–chitosan bioink enhance the bioink’s response to external forces. This was relevant for estimating its flow resistance under conditions analogous to those encountered during bioprinter extrusion. The angular frequency sweep revealed that the material exhibited a high storage modulus (G′), indicating predominantly elastic behavior (G′ > G″). This result indicated that while the formulation tended to maintain its structure and therefore potentially retain its shape after deposition, it was also essential to reduce viscosity during the process to facilitate its passage through the nozzle and prevent poor extrusion. The performance of the different samples in the experimental matrix was compared, and the dynamic viscosity was analyzed as a function of angular frequency (Figure 5A) to select the most suitable formulation for extrusion printing. In this analysis, sample C3–HS1 showed values closest to zero, interpreted as a tendency toward Newtonian behavior within the evaluated regime. Under applied stress, the viscosity tended to approach a more constant value while the force was maintained, a desirable condition for the extrusion process. Based on this evidence, it was decided to continue with C3–HS1, as it was considered the most viable formulation for forming simple geometries under controlled pressure and velocity conditions. Subsequently, the frequency sweep was performed exclusively for C3–HS1 and plotted on a logarithmic *x*-axis for ease of visualization (Figure 5B). To link the material’s behavior to operating conditions comparable to printing, the viscosity drop at *ω* = 0.1955 rad/s, considering the 25 mm diameter of the rheometer system, yielded approximate linear velocities of 3 mm/s, 6 mm/s, and 12 mm/s, corresponding to the points where the viscosity decreased experimentally. In the shear stress-versus-viscosity test (Figure 5C), it was observed that at relatively low pressures, viscosity increased, with a pronounced increase in the approximate range of 160 kPa to 175 kPa. However, at around 180 kPa, viscosity decreased to negative values, interpreted as a transition to a more favorable flow regime for extrusion. The response tended to stabilize at higher pressures and approached a more stable behavior around 225 kPa, although this value exceeded the maximum pressure available on the bioprinter. Therefore, it was concluded that it was necessary to adjust other printing parameters to reduce flow resistance, particularly by increasing the nozzle size. In the shear rate-versus-viscosity analysis (Figure 5D), a range of 10^−1^ s^−1^ to 10^3^ s^−1^ was identified as particularly active, corresponding to processes such as extrusion. This was used to evaluate the suitability of the hydrogel for 3D printing. The material appeared as shrinking layers under shear with shape recovery, consistent with predominantly elastic behavior and supporting its shape retention capacity after extrusion. Furthermore, viscosity increased with increasing levels of hydroxyapatite and silica, suggesting that silica contributed to the increased stiffness. This interpretation was supported by the findings of Fatma et al., who attributed this effect to silica’s ability to fill the pores of hydroxyapatite and enhance its mechanical properties. Therefore, the evaluated samples tended to resist flow, but retained the ability to maintain their shape after extrusion, which proved favorable for balancing extrusion and geometric stability in 3D extrusion printing [17,18,19].

Rheological characterization was used to screen collagen–chitosan bioink formulations doped with HA–SiO_2_ and to estimate their resistance to deformation under conditions relevant to extrusion-based printing. In oscillatory frequency sweeps, the bioinks exhibited a gel-like viscoelastic response, with the storage modulus exceeding the loss modulus over the evaluated angular frequency range (G′ > G″), indicating a predominantly elastic network with potential shape retention after deposition. The complex viscosity (η*) decreased markedly as angular frequency increased (0.1–100 rad·s^−1^) (Figure 5A), reflecting reduced resistance to deformation at higher oscillatory rates. Overall, these data emphasized the need to balance two competing requirements during printing: sufficiently low flow resistance to enable nozzle passage and sufficient structural integrity to preserve geometry after extrusion.

Based on the predefined selection criteria (Table 5), the best-performing formulations were shortlisted and compared using η* as a primary indicator of deformation resistance under oscillatory loading. Among the candidates shown in Figure 5, C3–HS1 consistently exhibited the lowest η* across most of the frequency domain and comparatively weaker frequency at intermediate-to-high ω, consistent with a stable response suitable for controlled extrusion. Therefore, C3–HS1 was selected as the most viable formulation to continue the remaining characterization steps. The frequency sweep of C3–HS1 was replotted on a logarithmic ω-axis (Figure 5B), confirming the progressive decrease in η* and the tendency toward a more stable, weakly frequency-dependent regime at higher frequencies (reduced frequency dependence rather than “Newtonian behavior”). In the raw rheometer export, occasional negative η* values were observed only at very low signal levels (low ω), consistent with measurements approaching the instrument torque sensitivity–noise floor. Because η* is reported as a magnitude, these negative values are non-physical; thus, they were retained in the raw dataset, but excluded from the processed plots and from quantitative interpretation (Figure 5B–D). This filtering was confined to the lowest-signal region and did not alter the observed trends or the comparative ranking among formulations.

Stress-controlled ramp testing was subsequently performed for C3–HS1 to further assess its resistance to deformation under increasing load (Figure 5C). The viscosity response showed a non-linear dependence on applied stress, consistent with a structured hydrogel network that resisted deformation at lower loads and transitioned toward facilitated flow as the imposed stress increased. Notably, the sharp decrease in apparent viscosity in the high-stress regime was interpreted as a transition to easier flow. Any subsequent negative viscosity value was considered artifactual and occurred beyond the reliable measurement range. This trend supported the need to reduce flow resistance through printing condition adjustments when pressure capacity was limited. Accordingly, printing was tuned by combining an increase in nozzle diameter to lower hydrodynamic resistance and reduce the required driving pressure and temperature control at the cartridge to decrease viscosity and facilitate extrusion. In parallel, printing speed was reduced to match attainable volumetric flow rate under the selected nozzle diameter and temperature, improving filament continuity and minimizing extrusion instabilities, consistent with the deformation trends observed rheological.

Viscosity trends evaluated over the extrusion-relevant deformation rate window (Figure 5D) indicated that the material response was most active approximately 10^−1^–10^3^ s^−1^, a range commonly associated with the extrusion processes. Overall, the rheological profile of C3–HS1 was consistent with a formulation that could deform under printing conditions while maintaining sufficient elastic character to recover structure after deposition. Increasing HA and SiO_2_ levels tended to increase viscosity and stiffness, supporting the interpretation that silica contributed to network reinforcement, in agreement with literature describing silica-mediated densification and strengthening mechanisms in HA-containing systems [17,18,19]. Collectively, the screened formulations exhibited mechanisms of a practical trade-off between flow resistance and post-extrusion stability, and C3–HS1 provided the most balanced profile for extrusion printing among the tested candidates (Figure 5).

Regarding temperature selection, rheological screening was carried out at 25 °C because precise thermal control was difficult to maintain with the rheometer configuration used and room-temperature testing provided a consistent basis for comparative selection among formulations. For bioprinting, 37 °C was selected for the cell deposition stage to match physiological conditions and support cell handling, whereas 45 °C was applied at the cartridge to take advantage of the expected temperature-dependent viscosity reduction, facilitating extrusion at lower resistance. Additionally, a 9 °C printing bed was implemented based on the consulted literature describing enhanced gelation/reticulation and improved shape fidelity of extruded bioinks under cooled substrates, thereby supporting post-deposition stabilization, while extrusion was enabled by a combined nozzle and cartridge temperature strategy.

### 2.4. Thermal Analysis

**Figure 6:**
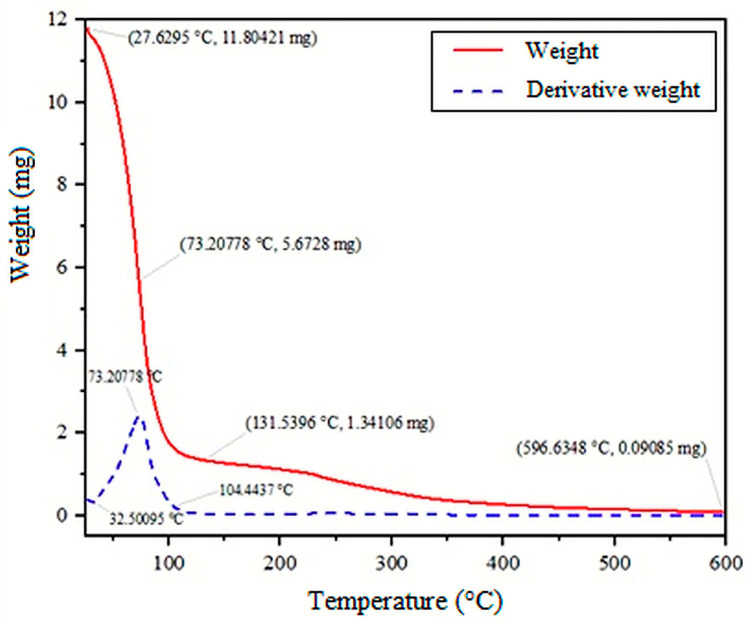
Thermogram of C3–HS1 bioink.

Figure 6 shows the relationship between temperature increase and bioink mass loss. The dashed line represents the first derivative of the mass percentage, that is, the rate of change as a function of temperature. The initial mass of the sample was 11.80421 mg at approximately 27 °C. The first change indicated by the derivative occurred between room temperature and 32 °C, signaling the beginning of water loss. Above 45 °C, the residual solvent began to evaporate and the sample started to lose mass more rapidly. The downward slope of the curve indicates mass loss associated with protein denaturation and the depolymerization of the polymer system. The peak near 80 °C signals the end of the water evaporation process. At around 600 °C, almost all the chitosan and collagen had degraded, leaving only the ceramic components, hydroxyapatite and silica, whose porous networks gave them high thermal stability [6,16,26,27].

The decomposition temperature of chitosan, the main organic component of the bioink, decreased upon interaction with collagen; however, the presence of hydroxyapatite and silica significantly increased the system’s thermal stability due to covalent interactions among chitosan, collagen, and the HA. Like molecular interactions between -OH, -COOH, and -NH2 on collagen chains and -OH and, -NH2 in chitosan were present, and there was an interaction between HA and amide I and amide II in collagen [21,28,29]. This thermal behavior enabled understanding of the bioink’s response to temperature changes during manufacture, storage, and use. This information was used to define the appropriate processing and preservation conditions [6,16,26,27].

### 2.5. Morphological Analysis

**Figure 7:**
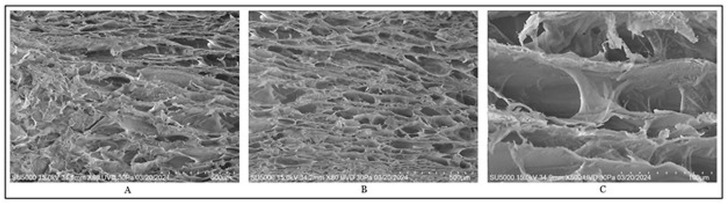
Scanning electron microscope images. (**A**) Sample C3–HS1, (**B**) magnification of 80× at a scale of 500 μm, (**C**) magnification of ×500 at 100 μm.

Figure 7 shows the images obtained by scanning electron microscopy, where the morphology of the marine collagen–chitosan bioink is shown once functionalized with silica-doped hydroxyapatite (C3–HS1), revealing a mainly rough and highly porous surface, with pores of varying sizes in an approximate range of 80 µm to 300 µm [6,16,22,27].

The presence of hydroxyapatite favored the formation of interconnected pores, attributed to its hexagonal crystalline structure, while silica further enhanced this porosity. As a result, when the ceramic compound was added to the chitosan–collagen hydrogel, the porosity increased significantly with moderate interconnections between pores, consistent with the findings reported by Rahman et al. This porous and interconnected architecture was considered relevant from a biomedical perspective, since the presence of adequate pores is essential for promoting blood flow and nutrient exchange in bone tissue [6,16,22,27].

### 2.6. Bioprinting Tests

An experimental matrix was designed and used, as presented in Table 1, based on the rheological properties of the marine collagen–chitosan bioink doped with hydroxyapatite and silica [6,13,16,26,27].

The bioprinting process was carried out using a pneumatic printhead and a 3 mL cartridge at a constant temperature of 37 °C, with the print bed maintained at 9 °C, using 14 G (1.5 mm)-diameter nozzles. Based on the reported rheological studies, a larger-diameter nozzle, physiological temperature, and a low extrusion speed were chosen. The increased nozzle diameter was intended to produce a continuous filament and reduce geometric fidelity loss during printing. A larger diameter decreases the dynamic viscosity and facilitates the material extrusion. Additionally, the increased temperature reduced viscosity and the bioink exhibited adequate performance under this condition, thanks to the presence of ceramic components [6,13,16,26,27].

As observed in Table 1, the printed structures maintained a more consistent shape when the printhead extruded the material slowly, in accordance with the rheological behavior shown in Figure 5. At a speed of 1 mm/s, the bioprinter experienced considerable mechanical stress and material consumption was excessive. Consequently, a speed of 5 mm/s was selected as the most suitable, along with a high pressure that allowed the desired shape to be obtained. Thus, the combination of 5 mm/s and 200 kPa was considered the ideal set of parameters, as it promoted a more viscous behavior of the bioink, which had predominantly behaved elastically in the rheological tests, and facilitated uniform material deposition [6,13,16,26,27].

The 14 G nozzle, considered the ideal sample, extruded a considerable volume of material. The same parameters were applied, varying only the nozzle diameter to smaller values. The results are shown in Table 2. It was observed that the printability limit was found to be circa 0.84 mm in diameter. At this size, the general shape was still preserved, although with less fidelity to the target geometry. The 1.25 mm nozzle, on the other hand, showed acceptable fidelity to the naked eye. To quantitatively verify the fidelity, images obtained with an optical microscope were used to measure the actual diameter of the deposited filament [6,13,16,26,27].

**Figure 8:**
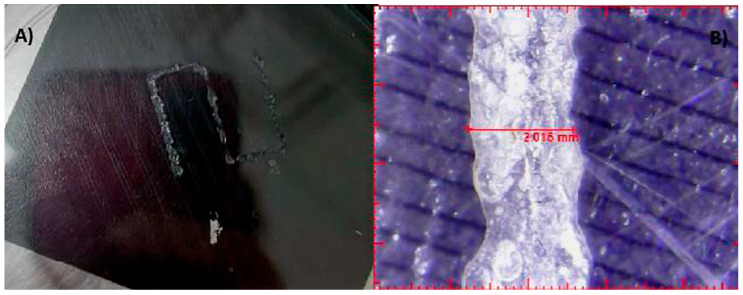
Final bioprinting test. (**A**) Bioink printing, (**B**) Filament printing fidelity.

The results of these measurements are summarized in Table 3. Fidelity was calculated as the ratio of the deposited filament diameter (1 mm/s), which exceeded to the nominal nozzle diameter by 31.4%, whereas under other evaluated conditions, it reached up to 4 times the nozzle diameter. The final bioprinting test consisted of increasing the system temperature to 45 °C while maintaining a constant speed of 5 mm/s, pressure of 200 Kpa, and nozzle size 1.5 mm (Figure 8) showed a fidelity impression with 2.015 mm, increasing 34.3% compared to the size of the nozzle used [6,13,16,26,27].

The results indicate that the hybrid formulation comprising marine collagen, chitosan, and silica-doped hydroxyapatite constitutes a structurally coherent and functionally robust bioink from a design perspective. Collagen extraction preserved key features of the triple-helix structure, while the HA–SiO_2_ ceramic phase maintained its chemical identity and provided a potentially osteoconductive inorganic component. The integration of both phases in the C3–HS1 formulation resulted in a hybrid polymer network where hydrogen bonding interactions between collagen and chitosan, along with the dispersion of the ceramic phase, yielded a cohesive hydrogel that was thermally stable within the operating range and possessed a porous, interconnected microstructure with pore sizes compatible with bone regeneration applications [6,13,16,26,27].

The rheological and process results showed that the bioink exhibited predominantly elastic behavior with shear thinning, which in principle favors pneumatic extrusion and shape retention after deposition. The same combination of high elasticity and higher viscosity induced by the HA–SiO_2_ phase resulted in a narrow printing window: it was necessary to work with high pressures, low printhead speeds, and relatively large-diameter nozzles to obtain continuous filaments and scaffolds with acceptable geometry. This allowed us to determine that the bioink can form stable three-dimensional structures with adequate porosity and an organic and ceramic composition of interest for bone tissue. Still, it also has significant limitations, such as the system’s sensitivity to small changes in printing parameters, filament overexpansion relative to the nominal nozzle diameter, and the lack, at this stage, of fine geometric fidelity and mechanical and biological validations [6,13,16,26,27].

## 3. Conclusions

This work demonstrated that the combination of marine collagen, chitosan, and a silica-doped hydroxyapatite ceramic phase (HA–SiO_2_) enables the formulation of a hybrid bioink with structural and rheological properties initially compatible with extrusion bioprinting processes. Collagen extraction from Nile tilapia (*Oreochromis niloticus*) skin preserved the triple-helix structure, as confirmed by FTIR spectroscopy. At the same time, characterization of the HA–SiO_2_ powder revealed the simultaneous presence of phosphate and carbonate groups as well as silicon-associated bands, supporting the formation of a doped, potentially osteoconductive inorganic phase.

The selected formulation, C3–HS1, integrated 85% chitosan and 15% collagen with a moderate HA–SiO_2_ fraction, forming a hybrid polymer network in which the organic phase provided gelation capacity and elasticity and the ceramic phase contributed rigidity and thermal stability. Thermogravimetric analysis confirmed that the bioink was stable within the temperature range required for bioprinting (30–45 °C). In contrast, morphological analysis revealed an interconnected porous architecture with pore sizes ranging from 80 µm to 300 µm, suitable for bone regeneration applications.

Rheological tests revealed predominantly elastic behavior (G′ > G″) in the linear range and a pseudoplastic response under shear, supporting the suitability of C3–HS1 for extrusion. However, the increased viscosity caused by the HA–SiO_2_ phase required the use of relatively high pressures and larger-diameter nozzles to achieve continuous extrusion. Bioprinting tests on the BIO X confirmed that the bioink was printable within a restricted parameter range, particularly at 5 mm/s and 200 kPa using 1.25 mm and 1.5 mm nozzles, yielding filaments with stable shape, but with diameter deviations from the nominal nozzle dimensions.

The C3–HS1 bioink is a viable initial formulation for generating three-dimensional scaffolds via extrusion bioprinting, with potential applications in bone tissue engineering due to its hybrid organic-ceramic composition, porous microstructure, and rheological properties. However, it was also established that the system requires further optimization to expand the printing window and improve geometric fidelity.

Three-dimensional bioprinting represents a disruptive technology that has transformed the biomedical and pharmaceutical sectors. Despite rapid growth and significant advances, several challenges associated with the use of marine-derived bioinks in 3D bioprinting remain unresolved or only partially addressed.

Currently, bioinks formulated from marine-derived biomaterials often lack the ability to form structurally stable constructs. Future research should prioritize enhancing the mechanical and biological properties of these bioinks, including improved stabilization of bioprinted structures and increased cell attachment and proliferation.

Limited resolution remains a significant challenge in 3D bioprinting. The ability to produce detailed three-dimensional structures using marine biomaterial-based bioinks is essential for broader adoption in the biomedical, pharmaceutical, and food industries. Consequently, efforts to improve the resolution of bioprinted constructs are necessary.

In conclusion, 3D bioprinting with marine biomaterial-based bioinks presents significant opportunities for biomedical, pharmaceutical, and food applications. Specifically, alginate, collagen, and gelatin—abundant in seaweed, fisheries, and aquaculture sidestreams—demonstrate considerable potential for future bioink development.

## 4. Materials and Methods

### 4.1. Synthesis of Materials

#### 4.1.1. Extraction of Marine Collagen by the Adjusted Ogawa Method

Collagen was extracted following the method of Ogawa et al. and adjusted by the BiomIT research group [23,24]. Four frozen Nile tilapia (*Oreochromis niloticus*) were purchased from a supermarket and kept within a cold chain: transport at −15 °C, storage between −22 °C and 0 °C, shelf display between 0 °C and 10 °C, and finally storage in domestic freezers at −18 °C. After thawing overnight, the skin was removed and the fillets were cut into 3 × 3 cm cubes.

The samples were first soaked in 200 mL of distilled water with 0.1 M NaOH (Fagalab, Mexico) under constant stirring (200 rpm) for 15 h. The following day, this was replaced with 400 mL of the same 0.1 M NaOH (Fagalab, Cerro Agudo, Mexico) solution and stirred for an additional 24 h. The material was then rinsed on a cloth-lined sieve until the pH reached 7. Next, it was left in 200 mL of distilled water with 0.5 M acetic acid for 3 days. The extract was decanted and centrifuged at 8000 rpm for 20 min. The supernatant was recovered and mixed with 200 mL of distilled water and 0.9 M NaCl (Fagalab, Mexico) and allowed to stand for 15 h. Finally, the solution was decanted onto the sieve, and the resulting precipitate was dialyzed for 25 min.

#### 4.1.2. Synthesis of Silicon Dioxide-Functionalized Hydroxyapatite

Silica-doped hydroxyapatite (50:50 composition of hydroxyapatite and crystalline silicon dioxide) was synthesized and doped at the Division of Sciences and Engineering of the University of Guanajuato. The hydroxyapatite was obtained by a hydrothermal method: 0.944 g of calcium nitrate tetrahydrate (Sigma-Aldrich, St. Louis, MI, USA) was dissolved in 20 mL of distilled water and stirred for 15 min, and 0.7923 g of diammonium phosphate (Sigma-Aldrich, USA) was dissolved in 20 mL of distilled water with constant stirring for 15 min. The phosphate solution was added to the calcium solution, and stirring was continued for an additional 15 min. The mixture was transferred to a furnace and treated in a hydrothermal reactor (NEWTRY, Columbus, OH, USA) at 140 °C for 4 h. After cooling, four centrifugal washes were performed (1500 rpm, 15 min each), dehydration was carried out at 50 °C for 18 h, and a heat treatment was applied at 1100 °C. Finally, the solid was ground in a mortar [30]. Silica was synthesized by mixing 40 mL of tetramethyl orthosilicate (Sigma-Aldrich, USA) with 40 mL of methanol (J.T. BAKER, Phillipsburg, NJ, USA), adding the methanol dropwise under stirring for 1 h. Subsequently, the pH was adjusted to 2 with HCl (J.T. BAKER, USA), and the solution was subjected to hydrothermal treatment at 90 °C for 24 h, dehydrated at 40 °C for 24 h, and ground in a mortar. The product was calcined at 600 °C for 6 h [31].

#### 4.1.3. Synthesis of Marine Collagen–Chitosan Bioink Doped with HA–SiO_2_

Chitosan hydrogels were synthesized with marine collagen, the bioink was prepared, and the experimental matrices on which the study was based were defined. The chitosan ratio was selected from Bautista et al. [27], with 90% high-molecular-weight chitosan (Sigma-Aldrich, USA) and 10% medium-molecular-weight chitosan for sol–gel synthesis. Collagen was added at the percentages specified in Table 4.

The base solution was prepared by mixing 100 mL of distilled water with 1 M acetic acid, and 25 mL of this solution was transferred to a 50 mL conical tube. Similarly, 900 mg of high-molecular-weight chitosan and 100 mg of medium-molecular-weight chitosan were weighed and dissolved in 1% acetic acid solution in 25 mL of distilled water. With the base solution prepared, 2 mL per sample was added, with the collagen percentages indicated in Table 4, and each preparation was stirred until the hydrogel was homogenized. The selected formulation was C3, with 85% chitosan and 15% collagen, as determined by the physicochemical characterization of Bautista et al. [27]. The percentages of silica-doped hydroxyapatite listed in Table 5 were added to sample C3, and the mixture was vortexed until complete homogeneity was achieved, yielding the bioink with all its components.

### 4.2. Physicochemical Characterization

#### 4.2.1. Rheology

Frequency sweep and stress ramp rheological tests were performed on an AR-2000 EX (TA Instruments, New Castle, DE, USA) rheometer to determine the viscous behavior of each formulation. The tests were conducted under the following conditions: angular frequency of 0.1–100 rad·s^−1^ in logarithmic mode, circular plate geometry of 25 mm, separation of 1 mm, and temperature of 25 °C (Standard Laboratory Conditions). Based on the results, the three samples with the best performance were selected according to the criteria in Table 5 and used to continue the remaining characterizations. Frequency sweep and stress ramp rheological tests were performed on an AR-2000 EX (TA Instruments, New Castle, USA) rheometer to characterize the viscoelastic behavior of fifteen formulations. Oscillatory frequency sweeps were conducted from 0.1 to 100 rad·s^−1^ in logarithmic mode using a 25 mm parallel-plate geometry, a 1 mm gap, and a temperature of 25 °C. The complex viscosity (η*) was obtained from the oscillatory response (η* = |G*|/ω). Based on the predefined selection criteria (Table 5), the three best-performing formulations were selected for the remaining characterizations [32,33,34].

#### 4.2.2. Chemical Composition

Chemical characterization was performed using FTIR spectroscopy on a Nicolet iS10 (Thermo Fisher Scientific, Waltham, MA, USA) instrument to identify functional groups in both the extracted materials and the hydrogels. Analyses were conducted in transmittance mode, using 64 scans over the 400–4000 cm^−1^ range and a resolution of 4 cm^−1^, and processed using OMNIC Paradigm 64 bits ™ software [22,32,33].

#### 4.2.3. Thermal Analysis

Thermogravimetric analysis was performed using an SDT-Q600 instrument (TA Instruments, New Castle, USA) with 5–10 mg samples. Mass loss was recorded as a function of temperature over 25 to 600 °C, with a heating rate of 5 °C/min under a nitrogen atmosphere [10,22,33].

#### 4.2.4. Morphological Analysis

Samples intended for morphological analysis by SEM (Hitachi SU-5000, Tokyo, Japan) were previously frozen for 48 h at −17 °C and subsequently lyophilized using a Freezone 2.5 instrument (SDT-Q600 instrument, New Castle, DE, USA) to remove solvents. Once dried, they were characterized using a Hitachi SU5000 microscope at 15 kV and magnifications of 80× and 500×. Pore size was determined by image analysis using ImageJ, version 1.54K [22,32,33].

### 4.3. Bioprinting Tests

The bioprinting tests of the scaffolds were carried out using extrusion, varying the printhead speed and extrusion pressure to identify the optimal printing conditions, while the process temperature was kept constant at 30 °C. The evaluated parameters were established according to the experimental matrix in Table 6, with speeds of 30 mm/s, 40 mm/s, and 60 mm/s and pressures ranging from 70 kPa to 160 kPa. The prints were performed on a BIO X platform (CellInk, Gothenburg, Sweden) using a 3 mL printhead and 1.5 mm (14 G) tips, using a pre-established design (2DModelDesignforBioX.stl) as a reference. During deposition, the print bed was maintained at 9 °C to promote scaffold solidification and stability. The resulting structures were evaluated using a 1000× digital microscope to determine the filament diameter [1,35,36].

## Figures and Tables

**Table 1:** Prints obtained with the C3–HS1 bioink based on the experimental matrix.

mm/s	100 kPa	150 kPa	200 kPa
1	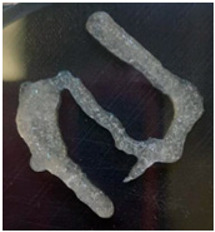	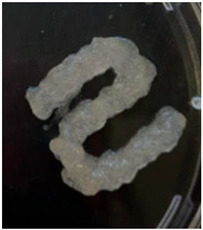	N/A
5	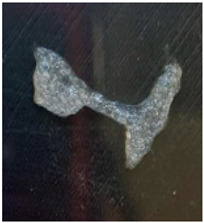	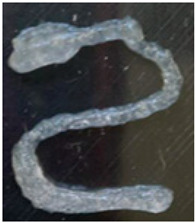	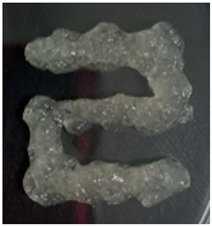
10	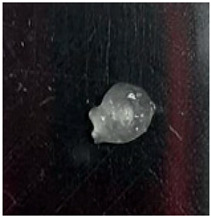	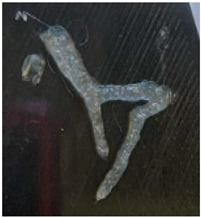	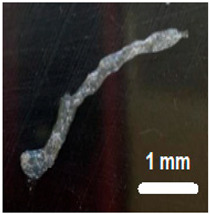

**Table 2:** Extrusion printing of marine collagen–chitosan bioink doped with hydroxyapatite and silica using different nozzle sizes.

Nozzle Size			
14 G	16 G	18 G	20 G
1.5 mm	1.25 mm	0.84 mm	0.61 mm
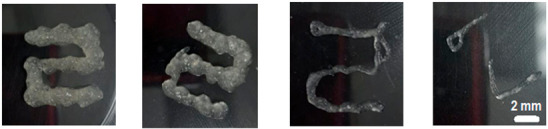			

**Table 3:** Filament and thickness obtained with the ideal printing parameters of the marine collagen–chitosan bioink doped with hydroxyapatite and silica.

Parameter				
Speed (mm/s)	5	5	5	1
Pressure (kPa)	200	200	150	100
Nozzle size (mm)	1.5	1.25	1.5	1.5
Fidelity	6.395 mm	5.329 mm	3.077 mm	1.971 mm

**Table 4:** Proposed experimental matrix for the synthesis of marine collagen–chitosan bioink.

Sample	Collagen	Chitosan
C1	5%	95%
C2	10%	90%
C3	15%	85%
C4	20%	80%
C5	25%	75%

**Table 5:** Proposed experimental matrix for the synthesis of marine collagen–chitosan bioink doped with HA–SiO_2_.

Sample	Chitosan	Collagen	Hydroxyapatite + Silica
C3–HS1	85%	15%	1%
C3–HS2			2%
C3–HS3			3%
C3–HS4			4%
C3–HS5			5%

**Table 6:** Printing parameters of the marine collagen–chitosan bioink doped with HA–SiO_2_.

Parameter	Level			
	1	2	3	4
Print speed (mm/s)	(30, 40, 60)	(30, 40, 60)	(30, 40, 60)	(30, 40, 60)
Extrusion pressure (kPa)	70	100	130	160

## Data Availability

The original contributions presented in this study are included in the article. Further inquiries can be directed to the corresponding author.

## Abbreviations

The following abbreviations are used in this manuscript.

FTIR	Fourier transform infrared
TGA	thermogravimetry
SEM	scanning electron microscopy
EP	extrusion printing
HA	hydroxyapatite
HA–SiO_2_	silica-doped hydroxyapatite
